# The evaluation of maximum condyle-tragus distance can predict difficult airway management without exposing upper respiratory tract; a prospective observational study

**DOI:** 10.1186/s12871-021-01253-5

**Published:** 2021-01-25

**Authors:** Hao Wu, Dandan Hu, Xu Chen, Xuebing Zhang, Min Xia, Xiaoqing Chai, Sheng Wang, Wei Zhang

**Affiliations:** 1 Department of Anesthesiology, First Affiliated Hospital of the University of Science and Technology of China, Anhui Provincial Hospital, 17 Lujiang Road, Hefei, 230000 Anhui China; 2 Department of Oncology, First Affiliated Hospital of the University of Science and Technology of China, Provincial Cancer Hospital, Hefei, Anhui China

**Keywords:** COVID-19 epidemic, Airway management, Difficult laryngoscopy

## Abstract

**Background:**

Routine preoperative methods to assess airway such as the interincisor distance (IID), Mallampati classification, and upper lip bite test (ULBT) have a certain risk of upper respiratory tract exposure and virus spread. Condyle-tragus maximal distance(C-TMD) can be used to assess the airway, and does not require the patient to expose the upper respiratory tract, but its value in predicting difficult laryngoscopy compared to other indicators (Mallampati classification, IID, and ULBT) remains unknown. The purpose of this study was to observe the value of C-TMD to predict difficult laryngoscopy and the influence on intubation time and intubation attempts, and provide a new idea for preoperative airway assessment during epidemic.

**Methods:**

Adult patients undergoing general anesthesia and tracheal intubation were enrolled. IID, Mallampati classification, ULBT, and C-TMD of each patient were evaluated before the initiation of anesthesia. The primary outcome was intubation time. The secondary outcomes were difficult laryngoscopy defined as the Cormack-Lehane Level > grade 2 and the number of intubation attempts.

**Results:**

Three hundred four patients were successfully enrolled and completed the study, 39 patients were identified as difficult laryngoscopy. The intubation time was shorter with the C-TMD>1 finger group 46.8 ± 7.3 s, compared with the C-TMD<1 finger group 50.8 ± 8.6 s (*p*<0.01). First attempt success rate was higher in the C-TMD>1 finger group 98.9% than in the C-TMD<1 finger group 87.1% (*P*<0.01). The correlation between the C-TMD and Cormack-Lehane Level was 0.317 (Spearman correlation coefficient, *P*<0.001), and the area under the ROC curve was 0.699 (*P*<0.01). The C-TMD < 1 finger width was the most consistent with difficult laryngoscopy (κ = 0.485;95%CI:0.286–0.612) and its OR value was 10.09 (95%CI: 4.19–24.28), sensitivity was 0.469 (95%CI: 0.325–0.617), specificity was 0.929 (95%CI: 0.877–0.964), positive predictive value was 0.676 (95%CI: 0.484–0.745), negative predictive value was 0.847 (95%CI: 0.825–0.865).

**Conclusion:**

Compared with the IID, Mallampati classification and ULBT, C-TMD has higher value in predicting difficult laryngoscopy and does not require the exposure of upper respiratory tract.

**Trial registration:**

The study was registered on October 21, 2019 in the Chinese Clinical Trial Registry (ChiCTR1900026775).

## Background

To control the pandemic and prevent novel coronavirus pneumonia nowadays, admitted patients are all required to wear masks to prevent the spread of epidemic disease. In order to predict difficult laryngoscopy, anesthesiologists must perform preoperative airway evaluation. The routine examination such as interincisor distance (IID), Mallampati classification, and upper lip bite test (ULBT) require a patient to remove his/her mask before opening the mouth while anesthesiologist conducting a close-up observation of the anatomical structure of the pharyngeal cavity and incisors and the process will undoubtedly increase the risk of nosocomial infection. Therefore, it is necessary to find new methods for protecting anesthesiologist from direct exposure of the upper respiratory tract accuracy and simplicity during preoperative airway evaluation.

The temporomandibular joint (TMJ) mobility plays a significant role in the grading of laryngoscopic exposure and the prediction of difficult laryngoscopy [1]. It is usually estimated by measurements including IID, Mallampati classification, as well as ULBT, which have certain predictive value of difficult airways with the accuracy and reliability still being limited [2]. Ultrasound measurement of the maximum movement distance of the condyle is thought to directly reflect the degree of TMJ mobility. It can be effectively used for preoperative airway assessment [3]. However, the method is slightly complicated due to the application of ultrasound.

Condyle-tragus maximal distance (C-TMD) can be used for preoperative airway assessment [4]. This method reflects the degree of TMJ mobility directly and can be simply completed while the patient wears a mask. However, compared with other indicators, such as Mallampati classification, IID, and ULBT, the value of the C-TMD remains unknown.

The purpose of this research was to observe the correlation and agreement between C-TMD and other valuable predictive indicators of difficult laryngoscopy in classifying laryngoscopy and predicting difficult laryngoscopy, and to calculate the predictive value of C-TMD. The number of intubation attempts and the time of intubation were also be recorded and calculated.

## Methods

This study was approved by the Ethics Committee of Anhui Provincial Hospital, the First Affiliated Hospital of the University of Science and Technology of China, under ethics approval number 2019KY No. 108. It was approved by the China Clinical Trial Registration Center under registration number ChiCTR1900026775. All trial participants were informed about the entire trial process and signed informed consent.

In this trial, we enrolled patients who underwent elective surgeries with endotracheal intubation under general anesthesia and were of ASA status I-III and 18–90 years of age. We excluded patients with no teeth, with maxillofacial injuries, inability to cooperate, a thyromental distance less than three fingers wide, or limited head and neck movement (less than 80 degrees).

**Fig. 1:**
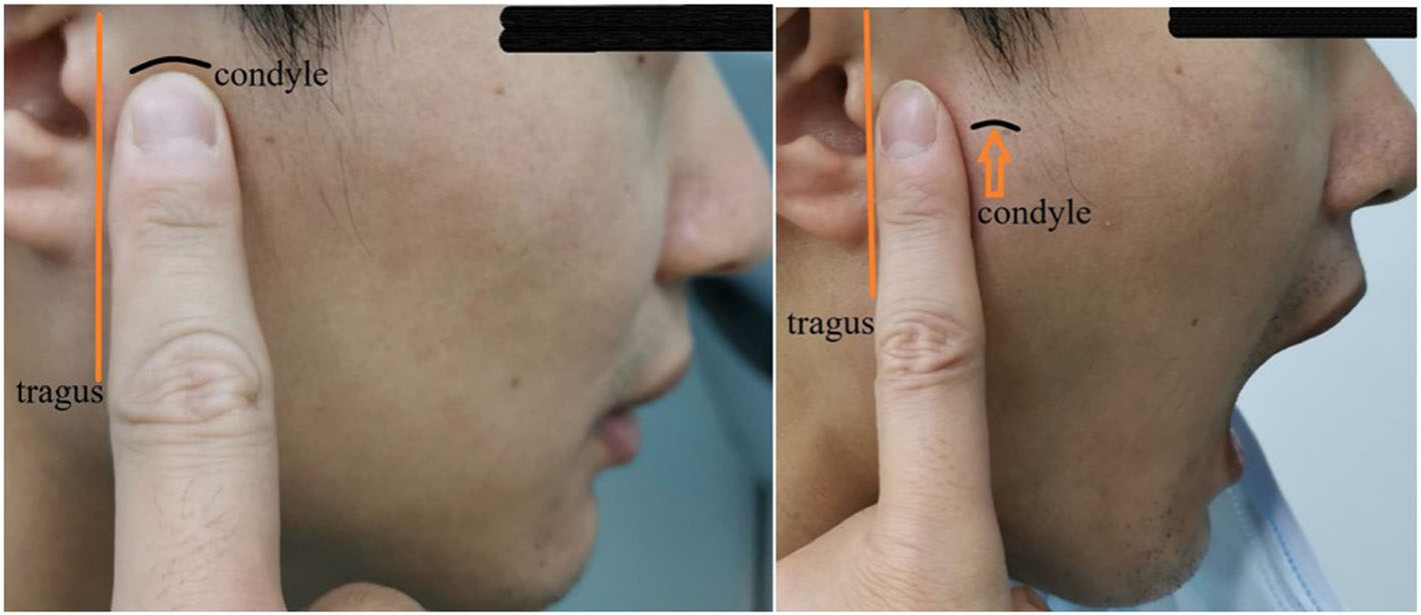
When opening the mouth as wide as possible, the condyle will move forward and down, the condyle-tragus maximal distance of this patient could accommodate one finger width without mask on

For all the patients enrolled in this study, during the preoperative examination 1 day before the operation or after the patient entered the preparation room on the day of the operation, an anesthesiologist who was skilled in the operative procedures used in this study examined whether the C-TMD could accommodate one finger width. The specific measurement procedure was as follows: The patient sat upright, and the examiner used the index fingers of both hands to locate the mandibular condyle of the mandible, instructed the patient to open the mouth as wide as possible, and felt that the condyle moved with the mouth opening movement. When the mouth opened as far as possible, the examiner then evaluated whether the distance between the condyle and the tragus could accommodate the width of one finger. The above measurement was repeated three times, and the maximum distance between the condyle and the tragus was taken (See Fig. 1 for details).

Later, another anesthesiologist, who was not aware of the evaluation results of C-TMD, measured other relevant indicators for airway evaluation. These indicators all indirectly reflected the degree of TMJ mobility:

Mallampati classification: The patient sat upright, opened the mouth wide, and extended the tongue to the maximum (no sound was made). The patient was then scored according to the pharyngeal structure that could be observed. Mallampati class > 2 was considered to be a predictive risk factor for difficult airways [5].

Interincisor distance (IID): The patient sat and opened the mouth as wide as possible, and then the doctor estimated IID with fingers. IID less than the width of three fingers was a predictive risk factor for difficult airways [6].

Upper lip bite test (ULBT) classification: The patient sat with the chin extending forward. The patient was asked to try his/her best to bite the upper lip with the lower incisors. According to the ability of the lower incisors to bite the upper lip, the test result was divided into three classes: Class 1: the lower incisors completely bit the upper lip above the vermilion border and completely covered the upper lip membrane; class 2: the lower incisors only bit half of the upper lip membrane and failed to reach the vermilion border; class 3: the lower incisor could not bite the upper lip. Classes 2 and 3 were the predictive risk factors for difficult airways [7].

All patients underwent routine electrocardiographic monitoring and induction of general anesthesia that started after the venous access was opened. The induction protocol utilized the following standardized recipe: midazolam 0.05 mg/kg, sufentanil 0.6 μg/kg, rocuronium 0.6 mg/kg and etomidate 0.3 mg/kg. An anesthesiologist with more than 3 years of experience, who was not aware of any preoperative airway evaluation results, conducted tracheal intubation with laryngoscopic exposure 3 min after bolus injection of rocuronium. According to the specific situation, either No. 3 or No. 4 laryngoscopy blades were used, and all patients took the head-up sniffing position. After intubation, the grading of all patients’ laryngoscopic exposure, the number of intubation attempts and the time of intubation were recorded. The time of intubation defined when the laryngoscope blade tip passed the incisors until confirmation of the first wave of carbon dioxide of the capnometer [8]. The Cormack-Lehane classification was used to grade laryngoscopic exposure, and observations of the structure of the larynx and the glottis were divided into four classes. Class 1: the glottis structure was fully exposed, and the front and back joint structure could be seen; class 2: the glottis was partially revealed, and the rear glottal joint structure could be seen; class 3: only the epiglottis was seen; class 4: neither the glottis nor epiglottis was visible, Classes > 2 were defined as difficult laryngoscopy [9]. In our institution, no more than 3 intubation attempts via the application of conventional laryngoscope blades were permitted to ensure patient safety, and the operating time for each attempt was no longer than 1 min. Before next intubation attempts, mask ventilation was used to ensure that the Spo2 was 98% or higher. If difficult airway appeared in the process, we followed the difficult airway treatment guidelines for the treatment and we also prepared conventional treatment tools such as a fiberoptic bronchoscope, laryngeal mask, and video laryngoscope.

### Reliability test

**Fig. 2:**
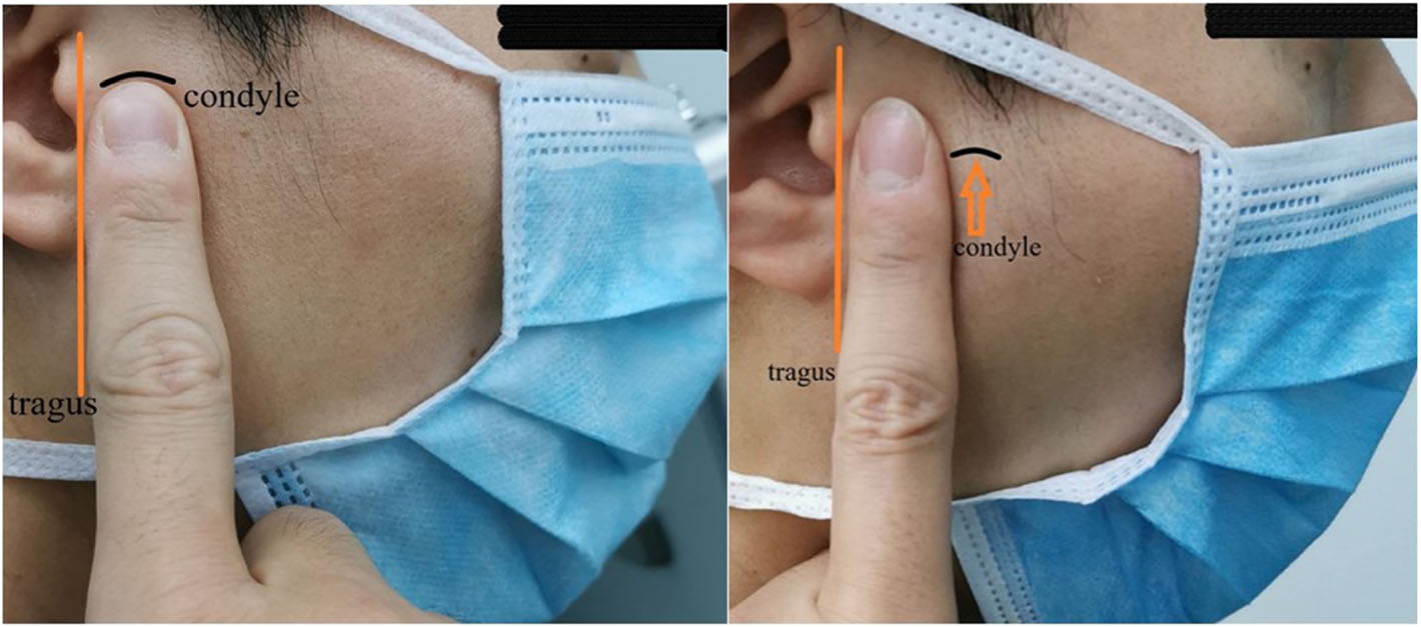
The condyle-tragus maximal distance of this patient could accommodate one finger width with mask on

To verify whether C-TMD accommodating the width of one finger can directly reflect the TMJ mobility and whether the method can accurately evaluate the condition when the patient wore protective equipment such as masks, we added two sets of reliability tests. We recruited 20 volunteers. All volunteers wore masks, and an anesthesiologist skilled in the experimental operation method evaluated whether the C-TMD of the volunteers could accommodate the width of a finger (See Fig. 2 for details). After the evaluation was completed, all volunteers took off their masks, and then another anesthesiologist skilled in the operation of this experiment, who was not aware of the previous measurement results, assessed whether the C-TMD of the volunteers could accommodate the width of a finger. The difference between the two evaluation results was compared. In addition, an anesthesiologist used ultrasound to measure the maximum condylar movement distance of all volunteers, that is, the degree of condylar mobility. We then analyzed the correlation between C-TMD and the degree of condyle mobility measured by ultrasound.

### Sample size

We conducted a pilot study of 50 patients for sample size assessment. In this pilot study, there were 19 patients with C-TMD < 1 finger, 31 patients with C-TMD>1 finger. The incidence of C-TMD<1 finger was 38%. After these 50 patients were divided into two groups according to whether C-TMD < 1 finger, the difference in intubation time that we observed between the C-TMD >1 finger groups (48.6 ± 7.2 s) and <1 finger groups (52.5 ± 8.4 s) was 4 s. In this study, using α = 0.05 and β = 0.1 and we found that a minimum of 176 participants were required.

### Statistical analysis

The SPSS 19.0 and MedCalc 19.2.0 statistical software packages were used. Measurement data were expressed as mean ± standard deviation ($\overline{\mathrm{x}}$ ± s), and ranked or categorical variables were expressed as frequency/ratio (n/%). For univariate comparison, the independent-sample t test, rank sum test, and chi-squared test were selected, according to specific circumstances. Spearman correlation analysis was used to analyze the correlation of variables, and the results of each predictor and laryngoscopic exposure were compared with the paired chi-squared test and internalagreement tests and kappa values were calculated. The receiver operating characteristic curve (ROC curve) was used to analyze the predictive value of each observed parameter to predict difficult laryngoscopy, expressed as the area under the curve (AUC) with its 95% confidence interval (95% CI), the comparison of 2 AUCs was performed using the DeLong’s test and the odds ratio (OR), specificity, and sensitivity of each index for predicting difficult laryngoscopy were calculated. A *P* < 0.05 indicated statistical significance.

## Results

### General information of patients and airway assessment results

Three hundred seventy-three patients were selected to be intubated under general anesthesia. Laryngeal mask airway management was performed in 58 patients, and surgery was temporarily canceled in 11 patients. Therefore, a total of 304 patients were successfully included in this study, including 137 male patients and 39 patients with difficult laryngoscopy. All patients were successfully intubated within 3 attempts. After group analysis of all patients according to whether they had difficult laryngoscopy, the differences of Mallampati classification, ULBT classification, IID, and the C-TMD between the two groups were statistically significant, while the differences of body mass index were not. Descriptive data of the patients and the airway assessment results are shown in Table 1.

**Table 1:** Comparison between the difficult and the non-difficult laryngoscopy group

Variable	Difficult laryngoscopy	Non-difficult laryngoscopy	*P* value
	*n* = 39	*n* = 265	
Sex (male/female, n)	26/13	111/154	0.003
Age (y)	57 ± 13	49 ± 16	< 0.001
Height (cm)	163 ± 7	165 ± 8	0.32
weight (kg)	62 ± 11	64 ± 12	0.52
Body mass index (kg/m^2^)	23.4 ± 3.7	23.4 ± 3.5	0.94
Mallampati classification (<3/>2grade, n)	5/34	128/137	< 0.001
ULBT (> 1/1grade, n)	33/6	148/117	< 0.001
IID<3 finger width (yes/no, n)	31/8	92/173	< 0.001
C-TMD < 1 finger width (yes/no, n)	35/4	81/184	< 0.001

Data are shown as the means±standard deviation or numbers. Difficult laryngoscopy was defined as a Cormack and Lehane grade > 2

All patient characteristics were compared using Mann-Whitney U test for continuous variables and χ^2^or Fisher exact test for categorical variables

*Abbreviations*: *ULBT* Upper lip bite test, *IID* interincisor distance, *C-TMD* condyle-tragus maximal distance

### The time of intubation and the number of intubation attempts of all predictors

The intubation time was shorter with the C-TMD >1 finger group 46.8 ± 7.3 s, compared to the C-TMD <1 finger group 50.8 ± 8.6 s (*P*<0.01). The intubation time differences of IID and Mallampati classification were statistically significant, while the ULBT were not. First attempt success rate was higher in the C-TMD >1 finger group 98.9% than in the C-TMD <1 finger group 87.1% (*P*<0.01). The intubation attempts differences of ULBT and Mallampati classification group were not statistically significant (see Tables 2 and 3).

**Table 2:** The number of intubation attempts of each predictor

		The number of intubation attempts 1/2/3(n)	*P* value
C-TMD	>1 finger	186/2/0	
	<1finger	101/9/6	<0.001
IID	>3finger	175/5/1	
	<3finger	112/6/5	0.023
ULBT	1 grade	120/2/1	
	>1 grade	167/9/5	0.143
Mallampati Test	<3 grade	124/9/0	
	>2 grade	163/2/6	0.064

The number of intubation attempts were compared using χ^2^or Fisher exact test for categorical variables

*Abbreviations*: *ULBT* Upper lip bite test, *IID* interincisor distance, *C-TMD* condyle-tragus maximal distance

**Table 3:** The time of tracheal intubation of each predictor

		The time of intubation	*P* value
C-TMD	>1 finger	46.8 ± 7.3 s	
	<1finger	50.8 ± 8.6 s	<0.01
IID	>3finger	47.1 ± 7.3 s	
	<3finger	50.1 ± 8.7 s	<0.01
ULBT	1 grade	47.5 ± 8.3 s	
	>1 grade	48.9 ± 7.8 s	0.146
Mallampati test	<3 grade	47.0 ± 7.9 s	
	>2 grade	49.3 ± 8.0 s	0.013

The time of intubation were compared using Mann-Whitney U test for continuous variables

*Abbreviations*: *ULBT* Upper lip bite test, *IID* interincisor distance, *C-TMD* condyle-tragus maximal distance

### Correlation of all predictors with intubation time and intubation attempts

The *r* values for all the predictors, such as the C-TMD, IID, ULBT, Mallampati test in comparison with the intubation time were 0.226 (*P*<0.001), 0.173 (*P*<0.01), 0.099 (*P =* 0.11), 0.171 (*P*<0.01), respectively, with the intubation attempts were 0.252 (*P*<0.001), 0.151 (*P*<0.01), 0.135 (*P*<0.05), 0.203 (*P*<0.01), respectively (see Table 4).

**Table 4:** Correlation analysis of each predictor (*n* = 304)

	C-L Levels (*r* /*P* value)	The number of intubation attempts (*r* /*P* value)	The time of intubation (*r* /*P* value)
C-TMD	0.317**/**< 0.001	0.252/< 0.001	0.226/< 0.001
IID	0.261**/**< 0.001	0.151/< 0.001	0.173/< 0.01
ULBT	0.266**/**< 0.001	0.135/< 0.05	0.099/0.11
Mallampati classification	0.213**/**0.002	0.203/< 0.001	0.171/< 0.01

Spearman correlation analysis was used for all correlations

*Abbreviations*: *ULBT* Upper lip bite test, *IID* interincisor distance, *C-TMD* condyle-tragus maximal distance, *C-L* Cormack-Lehane

### Comparison of preoperative predictors and the Cormack-Lehane levels

The *r* values of correlation between C-TMD, IID, ULBT, Mallampati classification and Cormack-Lehane Levels was 0.317,0.261,0.266 and 0.213 respectively (all *P* values were less than 0.01). Paired chi-square and agreement test showed that the C-TMD < 1 finger width had a significant *k* value (0.485) (see Tables 4 and 5 for details).

**Table 5:** Agreement test between each predictor and difficulty laryngoscopy (*n* = 304)

Predictors		Difficult laryngoscopy		κ value	95% CI
		NO	YES		
C-TMD	>1 finger	184	4		
	<1finger	81	35	0.485	0.286–0.612
IID	>3finger	173	8		
	<3finger	92	31	0.382	0.127–0.534
ULBT	1 grade	117	6		
	>1 grade	148	33	0.127	0.035–0.216
Mallampati Test	<3 grade	128	5		
	>2 grade	137	34	0.138	0.17–0.255

*Abbreviations*: *ULBT* Upper lip bite test, *IID* interincisor distance, *C-TMD* condyle-tragus maximal distance, *CI* confidence interval

### The predictive value of each predictor to predict difficult laryngoscopy

**Fig. 3:**
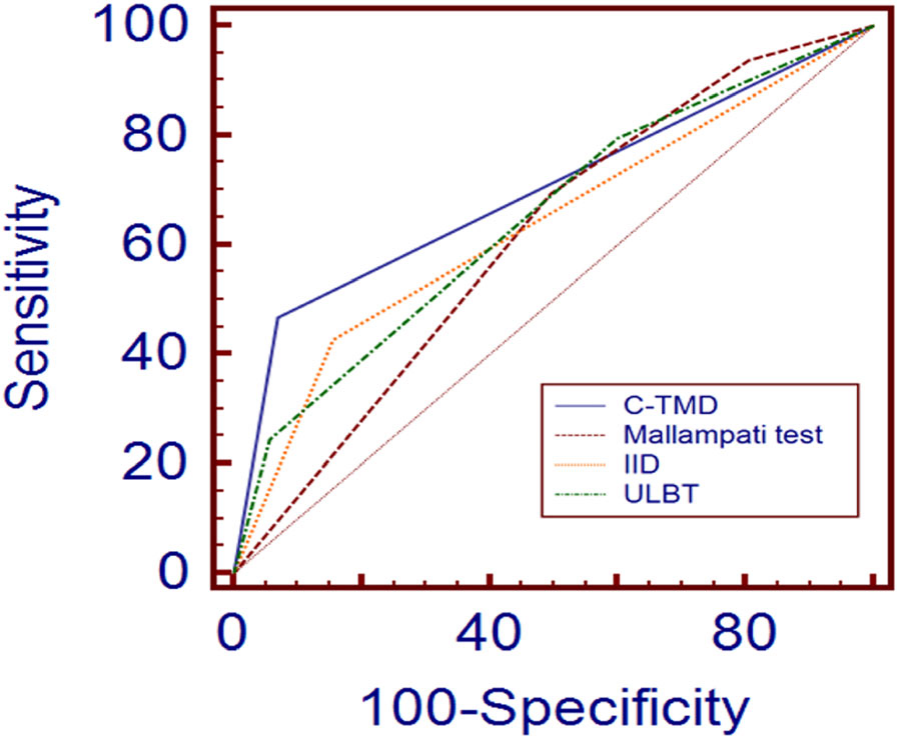
Receiver operating characteristic curve analysis of each predictor for predicting difficult laryngoscopy. IID indicates interincisor distance; ULBT, upper lip bite test; C-TMD, condyle-tragus maximal distance

The receiver operating characteristic (ROC) curve analysis showed that the AUC of the C-TMD, IID, Mallampati classification and ULBT classification for predicting difficult laryngoscopy were 0.699 (95% CI, 0.631 to 0.761), 0.637 (95% CI, 0.567 to 0.703), 0.613 (95% CI, 0.542 to 0.680) and 0.648 (95% CI, 0.579 to 0.714) respectively (compared with AUC = 0.5, *P* < 0.001 for all of them). Significant differences were observed between AUCs of the C-TMD to the other predictors (*P* < 0.001 for all the comparisons, See Fig. 3).

The OR value, sensitivity and specificity, positive predictive value and negative predictive value of each predictor were calculated. Among them, the predictive value of the C-TMD was the highest: OR value was 10.09 (95%CI: 4.19–24.28), sensitivity was 0.469 (95%CI: 0.325–0.617), specificity was 0.929 (95%CI: 0.877–0.964), positive predictive value was 0.676 (95%CI: 0.484–0.745), negative predictive value was 0.847 (95%CI: 0.825–0.865) (see Table 6 for details).

**Table 6:** Value of each predictor in predicting difficult laryngoscopy (*n* = 304)

Predictors	Odds ratio (95% CI)	Sensitivity (95% CI)	Specificity (95% CI)	PPV (95% CI)	NPV (95% CI)
C-TMD<1finger	10.09 (4.19–24.28)	0.469 (0.325–0.617)	0.929 (0.877–0.964)	0.676 (0.484–0.745)	0.847 (0.825–0.865)
IID < 3finger	3.54 (1.55–8.12)	0.429 (0.288–0.578)	0.845 (0.778–0.898)	0.467 (0.418–0.493)	0.824 (0.746–0.887)
ULBT>1grade	2.48 (1.02–6.02)	0.796 (0.657–0.898)	0.400 (0.322–0.482)	0.295 (0.174–0.324)	0.861 (0.735–0.923)
Mallampati classification >2grade	1.77 (0.78–3.91)	0.691 (0.546–0.817)	0.503 (0.422–0.584)	0.306 (0.276–0.371)	0.839 (0.813–0.908)

*Abbreviations*: *CI* confidence interval, *ULBT* Upper lip bite test, *IID* interincisor distance, *C-TMD* condyle-tragus maximal distance, *NPV* negative predictive value, *PPV* positive predictive value

### Results of reliability test

Twenty volunteers (14 males and 6 females) were successfully enrolled in the trial. There was no difference between the incidence rates of C-TMD < 1 finger width estimated by the two anesthesiologists (both were 5%). The maximum movement distance of the condyle measured by ultrasound was 12.6 ± 2.3 mm. Analysis of the correlation between whether the C-TMD < 1 finger width and the maximum movement distance of the condyle measured by ultrasound showed *r* = 0.91, and *P* < 0.001 (Spearman correlation analysis).

## Discussion

This study confirms that evaluating whether the C-TMD can accommodate the width of one finger can relatively effectively predict difficult laryngoscopy, and having significant correlation with intubation time and intubation attempts.

Significant differences of Mallampati classification, ULBT, IID and C-TMD have been found between difficult and non-difficult laryngoscopy group, proving all indicators to have certain value of prediction. The difference in age between the two groups was also significant. The most common age range of patients in the difficult laryngoscopy group was 44–70 years. This result was consistent with the recent study of Schnittker et al. [10] The intubation attempts and time significant differences of whether the C-TMD can accommodate the width of one finger reveal that C-TMD < 1 finger width indicates prolonged intubation time and increased number of intubation attempts compared with C-TMD > 1 finger width. The reason is because a patient presenting grade of 3 or 4 in the Cormack-Lehane grade is known to be in high risk of several intubation attempts or intubation failure [11].

The airway evaluation before anesthesia mainly includes accurate measurement and finger width estimation. Finger-width estimation is more widely used because of its simplicity in large-scale top-tiered hospitals with high surgery volume. Yao et al. [3] used ultrasound to measure the distance moved by the condyle before and after the opening of the mouth to evaluate the degree of condyle mobility and applied it to the prediction of difficult laryngoscopy. The resulting AUC value of the ROC curve was 0.934, which is higher than those of the accurate measurement methods IID, ULBT grading, and Mallampati classification. The current method has used the tragus as a reference line and used the width of the finger to estimate the maximum distance between the condyle and the tragus. This method can avoid the constraints of objective conditions, such as the availability of ultrasound, and is more convenient. The AUC value of C-TMD was 0.699, which was higher than the AUCS from the finger-width estimation of IID, ULBT grading, and Mallampati classification. These results were basically consistent with those of Yao et al.

C-TMD had the highest agreement with the laryngoscope classification. Reliability testing results showed that C-TMD was highly correlated with the maximum movement distance of the condyle measured by ultrasound. The maximum movement distance of the condyle measured by ultrasound can directly reflect TMJ mobility. Thus C-TMD can directly reflect the degree of TMJ mobility as well. The process of laryngoscopic exposure is actually the process of mandibular opening and forward movement, in which the condyle is the pivot point of the entire movement [12]. The wider the range of motion of the condyle, the greater the potential for mandibular movement. Sójka et al. [13] also showed that the degree of TMJ mobility was closely correlated with the range of motion of the condyle at the maximum mouth opening. Taking the tragus as a reference, C-TMD can reflect the maximum mobility of the condyles. Therefore, C-TMD < 1 finger width may be an independent risk factor for difficult laryngoscopy.

The results of this research has showed that the specificity of this predictive index of C-TMD < 1 finger width was 0.929, and the positive predictive value was 0.676, higher than those of other indices, indicating that the misdiagnosis rate and missed diagnosis rate of this index were lower than those of other related indicators. In predicting difficult laryngoscopy, the indicator of IID < 3 finger width, which is used most frequently in our clinical practice, only had a positive predictive value of 0.467, in line with the findings of Chhina et al. [14] These data further support the advantage of C-TMD < 1 finger width in predicting difficult laryngoscopy.

From the perspective of intubation time, intubation attempts, correlation and predictive value, the main reasons for the unsatisfactory performance of ULBT may be its misdiagnosis rate is high. Many patients without difficult laryngoscopy are misdiagnosed because of higher ULBT classes [15]. Our results were consistent with this observation and showed that the sensitivity of high ULBT class was 0.796, while its positive predictive value was only 0.295. The reason that patients could not bite upper lip above the vermilion border with lower incisors might be thick lips rather than reduced TMJ mobility. Therefore, whether lip thickness is partly responsible for the low positive predictive value of ULBT classification needs to be further investigated.

At present, COVID-19 has broken out all over the world. While adopting protection for ourselves and our patients, we can improve the traditional diagnosis and treatment methods to reduce the cross-infection rate between medical staff and patients [16]. For patients undergoing elective surgery in the new environment, preoperative airway assessment is essential, but the examination may bring anesthesiologists plenty of unknown risks. It is meaningful to find a way to balance the effectiveness and safety of airway assessment. The assessment of C-TMD can be completed with a high predictive value even if the patient wear personal protective equipment such as mask. During this pandemic, it can be used as a simple method to reduce the exposure of the upper respiratory tract for predicting difficult laryngoscopy instead of IID, Mallampati classification, and ULBT. What’s more, if C-TMD predicted a prolonged intubation and increased number of intubation attempts, the exposure time to the open mouth should decrease by the application of advanced equipment such as a video laryngoscope.

This study still has some limitations. First, in the perspective of methodology, the method of evaluating the condyle mobility depended on the estimation using finger width. Whether it will have lower predictive value than accurate measurement especially in obese patients still needs to be confirmed. Second, the sample size of this study was not big enough that we cannot analyse difficult intubation due to its low incidence. Third, the difference in intubation time between the C-TMD>1 finger and <1 finger group is 4 s, which is clinically insignificant, although statistically significant. In addition, 4 s is very short to show the effectiveness of a technique for assessing the airway. However, according to the principles of COVID-19 airway management, “swiftness” is very important [17]. Therefore, it may make sense during periods of specific respiratory disease epidemics.

As we know, the most useful tool to assess difficult airway management is the el ganzouri score, which is more comprehensive than single evaluation [18]. Therefore, the assessment of C-TMD is useful only when an appropriate full evaluation is not available (such as viral epidemic situation). The width of the index finger of a normal adult is approximately 1.2 cm. Whether this means that C-TMD less than 1.2 cm is a high-risk factor for difficult laryngoscopic exposure still needs to be explored through visualization techniques such as ultrasound. In the next study, we will enlarge sample size to research its value to predict difficult intubation and use ultrasound to locate the condyle to measure C-TMD, in order to calculate the error rate of finger positioning, eliminate the impact of individual finger-width differences on the prediction results, and compare the advantages and disadvantages of ultrasound positioning and finger positioning.

## Conclusions

In summary, compared with the IID, Mallampati classification and ULBT, the C-TMD has higher value in predicting difficult laryngoscopy and does not require the exposure of upper respiratory tract.

## Data Availability

The datasets used and analysed during the current study are available from the corresponding author on reasonable request.

## Abbreviations

ASA: American Society of Anesthesiology
BMI: Body mass index
IID: Interincisor distance
ULBT: Upper lip bite test
C-TMD: Condyle-tragus maximal distance
